# Stoichiometry of Rtt109 complexes with Vps75 and histones H3-H4

**DOI:** 10.26508/lsa.202000771

**Published:** 2020-09-10

**Authors:** Noushin Akhavantabib, Daniel D Krzizike, Victoria Neumann, Sheena D’Arcy

**Affiliations:** 1 Department of Chemistry and Biochemistry, The University of Texas at Dallas, Richardson, TX, USA; 2 Department of Biochemistry and Molecular Biology, Colorado State University, Fort Collins, CO, USA

## Abstract

Histone acetylation is one of many posttranslational modifications that affect nucleosome accessibility. Vps75 is a histone chaperone that stimulates Rtt109 acetyltransferase activity toward histones H3-H4 in yeast. In this study, we use sedimentation velocity and light scattering to characterize various Vps75–Rtt109 complexes, both with and without H3-H4. These complexes were previously ill-defined because of protein multivalency and oligomerization. We determine both relative and absolute stoichiometry and define the most pertinent and homogeneous complexes. We show that the Vps75 dimer contains two unequal binding sites for Rtt109, with the weaker binding site being dispensable for H3-H4 acetylation. We further show that the Vps75–Rtt109–(H3-H4) complex is in equilibrium between a 2:1:1 species and a 4:2:2 species. Using a dimerization mutant of H3, we show that this equilibrium is mediated by the four-helix bundle between the two copies of H3. We optimize the purity, yield, and homogeneity of Vps75–Rtt109 complexes and determine optimal conditions for solubility when H3-H4 is added. Our comprehensive biochemical and biophysical approach ultimately defines the large-scale preparation of Vps75–Rtt109–(H3-H4) complexes with precise stoichiometry. This is an essential prerequisite for ongoing high-resolution structural and functional analysis of this important multi-subunit complex.

## Abstract (precis)

The work determines the relative and absolute stoichiometry of a 5-chain protein complex involved in histone chaperoning and acetylation. Using sedimentation velocity and light scattering, it uncovers a dynamic equilibrium of complex self-association.

## Introduction

The nucleosome is the repeating unit of chromatin and consists of 146 bp of DNA wrapped around a histone octamer. The histone octamer is composed of two copies of histone H2A-H2B dimer and one copy of histone (H3-H4)_2_ tetramer. Within the octamer, the histones interface through four-helix bundles between the two copies of H3 (H3 and H3′), as well as H4 and H2B (Luger et al, 1997; McGinty & Tan, 2015). Several lysine residues in the histones are acetylated by enzymes that transfer an acetyl group from an acetyl-CoA cofactor. This acetylation modulates the chromatin structure to regulate processes such as DNA replication, repair, and transcription (Shahbazian & Grunstein, 2007; Bannister & Kouzarides, 2011).

In yeast, Rtt109 is an acetyltransferase that modifies various H3-H4 lysine residues, including H3 K9, K27, and K56 (Schneider et al, 2006; Fillingham et al, 2008; Radovani et al, 2013). H3-K56 acetylation is important in fungal pathogenicity, making Rtt109 an attractive antifungal therapeutic target (Wurtele et al, 2010; Dahlin et al, 2014). The position of acetylation is important as it determines the specific downstream effect. H3-K9Ac and H3-K27Ac, for example, influence transcription, whereas H3-K56Ac is additionally associated with DNA replication and repair (Roh et al, 2005; Recht et al, 2006; Chen et al, 2008; Li et al, 2008; Creyghton et al, 2010). The regulation of Rtt109 activity and selectivity is purportedly modulated by two histone chaperones, Vps75 and Asf1 (Driscoll et al, 2007; Tsubota et al, 2008; Kuo et al, 2015). Here, we study the complex between Vps75, Rtt109, and H3-H4.

Vps75 belongs to the nucleosome assembly protein family of histone chaperones (Selth & Svejstrup, 2007). It is composed of two chains that pack together through a head-to-tail coiled coil, with each end of the coiled coil also containing a globular domain (Tang et al, 2008). The Vps75 dimer can also dynamically self-associate to form a homo-tetramer (a dimer of dimers) depending on the protein and salt concentration (Bowman et al, 2014). Vps75 binds H2A-H2B and H3-H4 with nanomolar affinity, and this interaction interferes with Vps75 tetramerization but not with the four-helix bundle interface of (H3-H4)_2_ (Park et al, 2008; Hammond et al, 2016). To date, the complex between Vps75 and H3-H4 has been refractory to high-resolution structural studies.

Vps75 also binds Rtt109 tightly, and several crystal structures of this complex are available (Albaugh et al, 2010; Su et al, 2011; Tang et al, 2011). These crystal structures show either a 2:1 or a 2:2 complex and identify an Rtt109 loop important for interaction (Su et al, 2011; Tang et al, 2011). Vps75 enhances Rtt109 acetyltransferase (*k*_*cat*_ increased ∼100-fold) and favors H3-K23Ac and H3-K27Ac (Berndsen et al, 2008; Fillingham et al, 2008; Keck & Pemberton, 2011). This enhancement is 20-fold greater than the enhancement with Asf1, which favors H3-K56Ac (Tsubota et al, 2008). A structural model with both Vps75 and Asf1 suggests these preferences are driven by fuzzy electrostatic interactions between the negative C-terminal tail of Vps75 and the positive N-terminal tail of H3 (Danilenko et al, 2019).

Our goal is to develop a more complete understanding of Rtt109 regulation by characterizing the complex between Vps75 and Rtt109 both with and without H3-H4. Given the various crystal structures of Vps75 with Rtt109, and the ability of both Vps75 and H3-H4 to oligomerize, we focus on determining relative and absolute stoichiometry. We use various strategies to optimize the purity, yield, homogeneity, and solubility of relevant complexes. Our protocols will facilitate future high-resolution structural approaches. Ultimately, we reveal that Vps75, Rtt109, and H3-H4 form a 4:2:2 complex that is composed of two 2:1:1 sub-complexes. These sub-complexes come together through the four-helix bundle of the (H3-H4)_2_ tetramer.

## Results

### Vps75 homodimer contains two unequal Rtt109-binding sites

**Figure 1.**
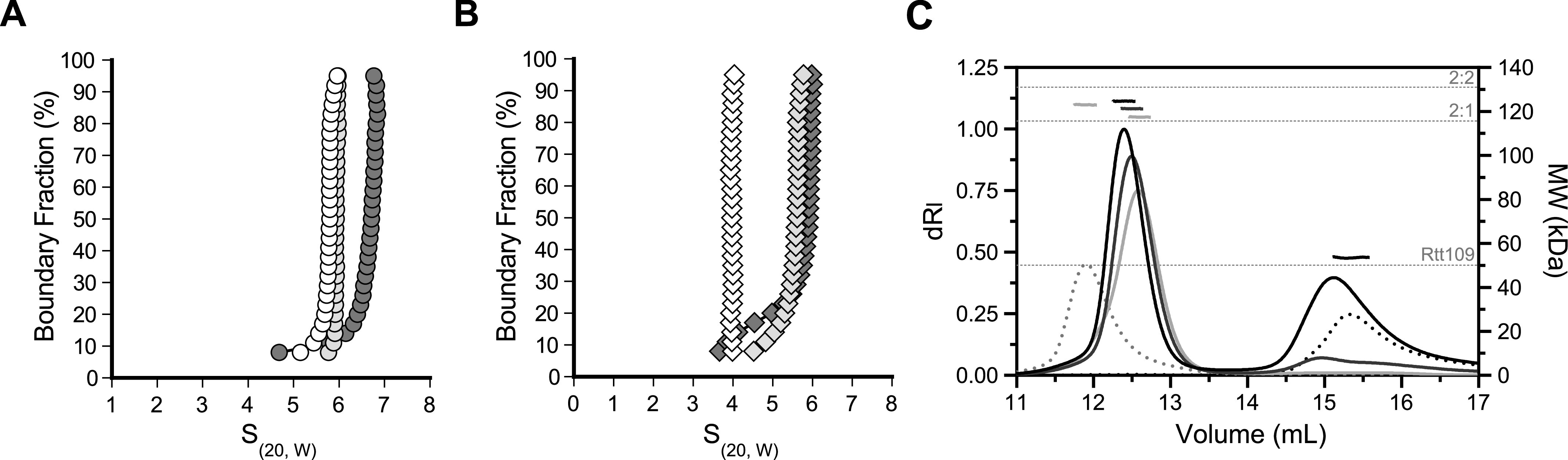
Stoichiometry of Vps75–Rtt109 complexes. **(A, B)** van Holde–Weischet analysis of sedimentation velocity experiments of 7.5 μM Vps75 with 0, 0.5, and 1.0 equivalent (white, light grey, and dark grey, respectively) of Rtt109 at 150 mM NaCl (A, circles) or 300 mM NaCl (B, diamonds). Similar experiments for Rtt109 and H3-H4 are shown in Fig S1. **(C)** SEC-MALS of 10 μM Vps75 (dotted grey), 10 μM Rtt109 (dotted black), and 10 μM Vps75 with 5, 10, or 20 μM Rtt109 (light grey, dark grey, and black, respectively). Horizontal dashed lines indicate theoretical molecular weights of labeled complexes.

**Figure S1.**
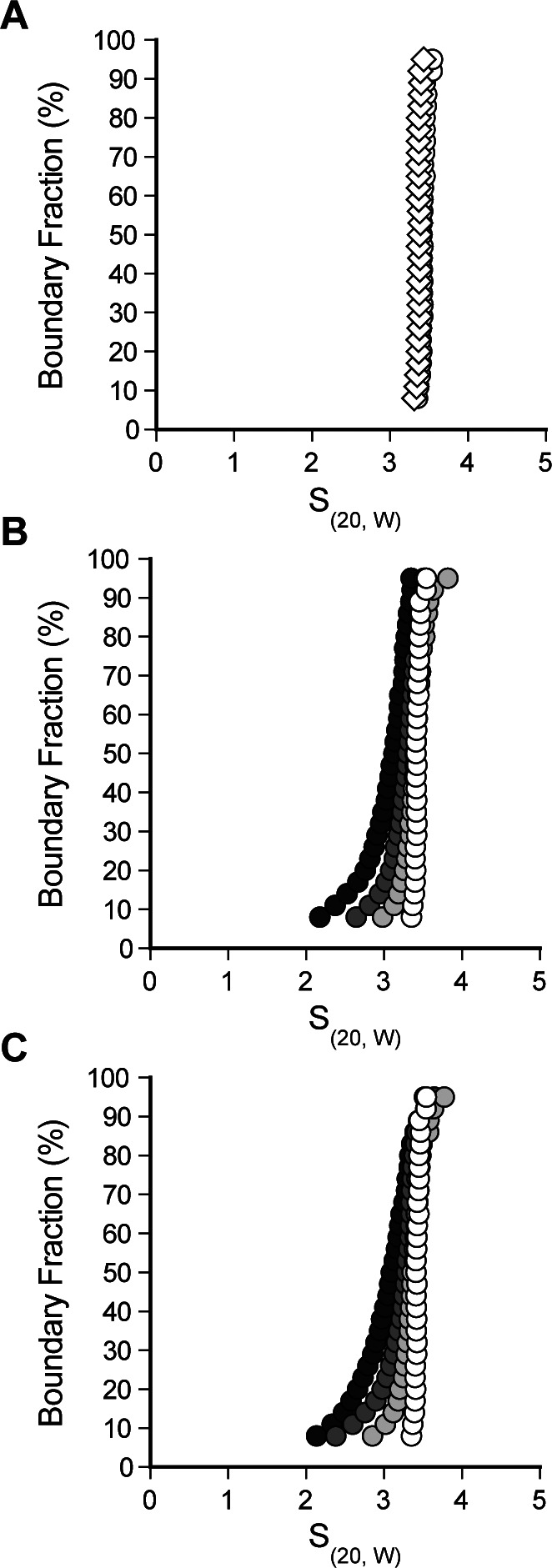
Rtt109 does not stably interact with H3-H4 or H3_DM_-H4. **(A, B, C)** van Holde–Weischet analysis of sedimentation velocity experiments of Rtt109 alone (A) or with 0 to 2 equivalents of H3-H4 (B) or H3_DM_-H4 (C). Rtt109 alone is shown in white, and Rtt109 with 0.5, 1, or 2 equivalents of histones is shown in light grey, dark grey, and black, respectively.

Similar to many proteins in the Nap family, Vps75 adopts dimeric and tetrameric assemblies depending on the ionic strength of the buffer (McBryant & Peersen, 2004; Bowman et al, 2014; Sarkar et al, 2020). Vps75 also interacts with Rtt109 with reported stoichiometries of 2:1 and 2:2 (Su et al, 2011; Tang et al, 2011). To determine the influence of ionic strength on this stoichiometry, we have performed sedimentation velocity analysis with mixtures of individually purified proteins (Table S1). In this setup, we detected Vps75 tetramers and dimers at 5.8 and 4.0 S, respectively (Fig 1A and B, white). We analyzed these Vps75 assemblies with various equivalents of Rtt109. At 150 mM NaCl, addition of 0.5 equivalents did not increase sedimentation, whereas the addition of one equivalent increased sedimentation to 6.7 S (Fig 1A, grey). An increase was not observed at 0.5 equivalents as Rtt109 splits a Vps75 tetramer into two dimers, as shown by others (Hammond et al, 2016). These data suggest that the 6.7 S species is a 2:2 complex at 150 mM NaCl. By contrast, at 300 mM NaCl, addition of 0.5 or 1 equivalent both increased sedimentation to 5.9 S (Fig 1B, grey). Addition of one equivalent also resulted in the appearance of a tail tending toward 3.2 S, indicative of some unbound Rtt109 (Fig S1A). This shows that the 5.9 S species is a 2:1 complex at 300 mM NaCl. Thus, the Vps75 dimer can bind two copies of Rtt109 at 150 mM NaCl and only one copy at 300 mM NaCl.

Table S1 Summary of sedimentation velocity samples.

To ascertain the absolute stoichiometry of the complexes, we performed size exclusion chromatography coupled to multi-angle light scattering (SEC-MALS) at 150 mM NaCl (Fig 1C). The Vps75 tetramer eluted at 11.9 ml, and Rtt109 eluted at 15.3 ml. Mixtures of Vps75 with 0.5, 1, or 2 equivalents of Rtt109 eluted between these values, reinforcing that Rtt109 binding splits a Vps75 tetramer into two dimers. At 0.5 equivalents, the measured molecular weight (MW) was 117.5 ± 0.07 kD, indicating a 2:1 complex (theoretical MW of 115.7 kD). When Rtt109 is increased to 1.0 and 2.0 equivalents, the MW did increase but remained below a fully saturated 2:2 complex. Unbound Rtt109 was also visible in the chromatogram. These data indicate that while two copies of Rtt109 can bind a Vps75 dimer, the second copy binds with less affinity than the first and is lost during chromatographic separation. Two unequal binding sites were previously suggested by sedimentation equilibrium experiments (Park et al, 2008). A second copy of Rtt109 will not bind the Vps75 dimer at either low protein or high salt concentration.

### Stoichiometry of co-purified Vps75-Rtt109 is 2:1

**Figure 2.**
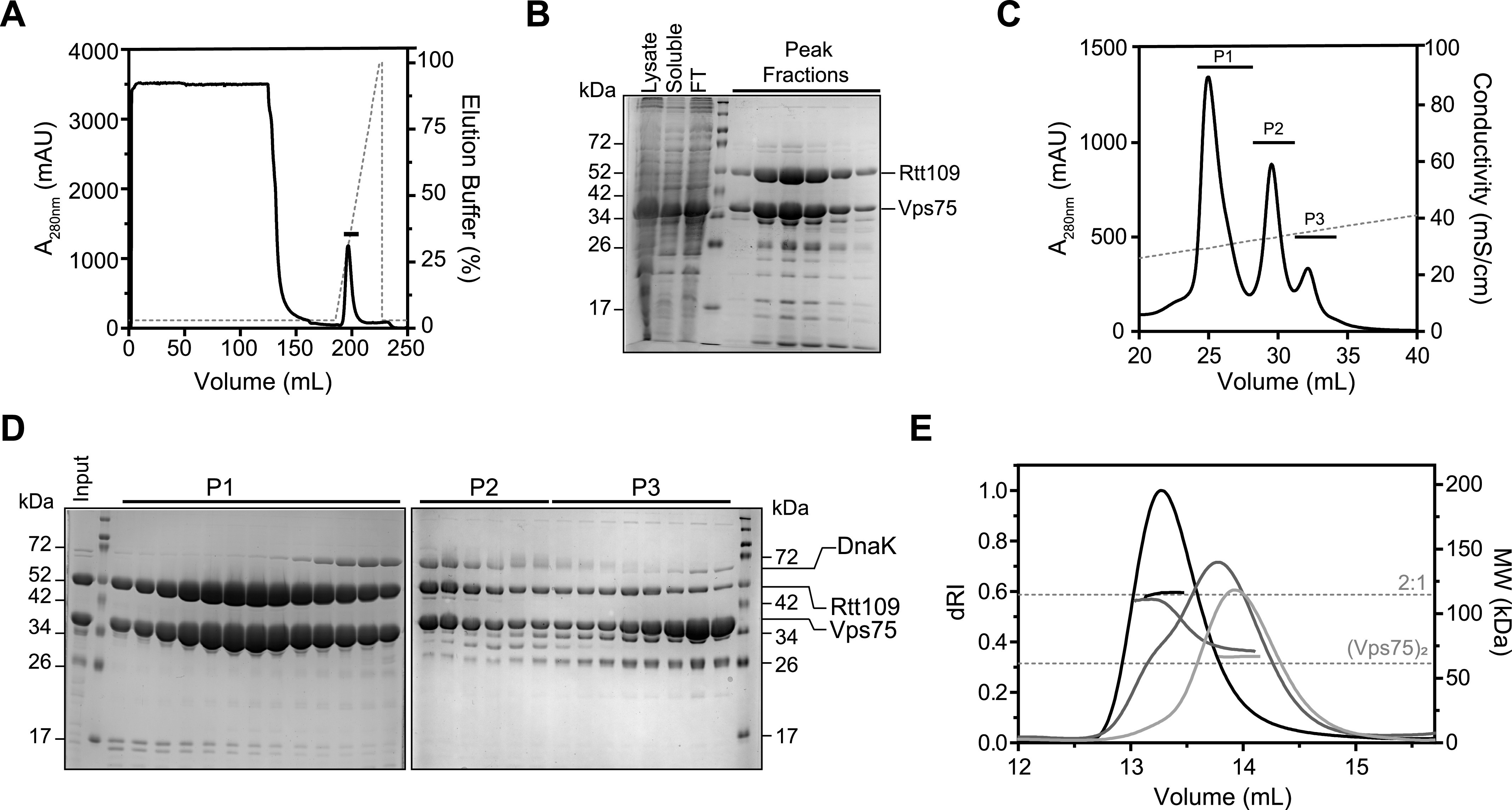
Co-purification of Vps75–Rtt109. **(A, B, C, D)** Purification of co-expressed his–Rtt109 and Vps75 by Ni^2+^-affinity (A, B) and anion exchange (C, D) chromatography. FT is flow-through. **(E)** SEC-MALS of major peaks at 300 mM NaCl from anion exchange. P1, P2, and P3 are shown in black, dark grey, and light grey, respectively. Horizontal dashed lines indicate theoretical molecular weights of labeled complexes. Optimization of a MgATP wash is shown in Fig S2.

Having determined the stoichiometry of reconstituted Vps75–Rtt109 complexes, we sought to measure the stoichiometry of a co-expressed complex. Co-expression is more convenient and, in this case, produces higher yields than Rtt109 alone. Different expression and purification strategies can cause different stoichiometries, and our initial attempts using reported protocols resulted in heterogeneous samples (Tang et al, 2011). The first step in purification is Ni^2+^-affinity chromatography via an N-terminal his tag on Rtt109 (Fig 2A). We found that a 5-ml HisTrap HP column (GE Healthcare) is saturated by complex from 6 liters of *Escherichia coli* culture. Increasing column volume or rerunning the flow-through did not improve the yield. Notably, Vps75 was detected in the column flow-through, suggesting it was overexpressed compared with Rtt109, or that Rtt109 was depleted during purification (Fig 2B). The second step of purification is anion exchange, and three peaks containing Vps75 and Rtt109 eluted at 200–400 mM NaCl (Fig 2C). The most intense peak (P1) contained an ∼70 kD contaminant, whereas the other two peaks (P2 and P3) had different relative amounts of Vps75 and Rtt109 (Fig 2D).

**Figure S2.**
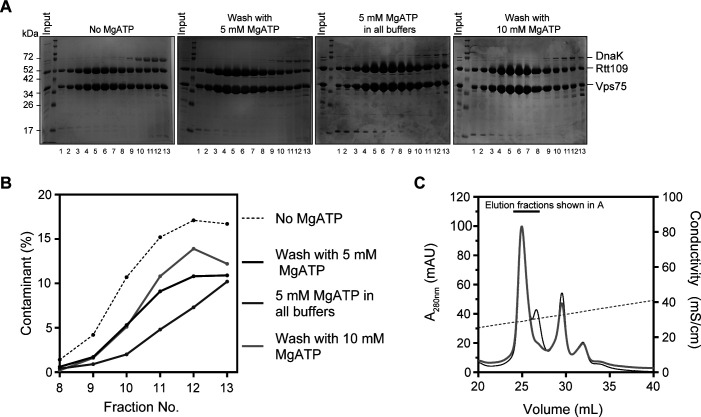
Removing DnaK from co-expressed Vps75–Rtt109 using MgATP. **(A)** SDS–PAGE of anion exchange elution fractions under different conditions. From left to right, no MgATP, 5 mM MgATP wash during Ni^2+^-affinity chromatography, 5 mM MgATP in lysis and chromatography buffers, and 10 mM MgATP wash during Ni^2+^-affinity chromatography. The latter two conditions cause a dramatic increase in system pressure. **(B)** Quantification of the amount of DnaK contamination in each anion exchange fraction. **(C)** Anion exchange chromatogram of Vps75–Rtt109 without MgATP (black) and with a 5 mM MgATP wash (grey).

We improved the purification of co-expressed Vps75–Rtt109 by identifying and removing the ∼70 kD contaminant. In-gel digestion followed by mass spectrometry showed it to be DnaK, an ATP-dependent, bacterial, folding chaperone. We recovered peptides for 75% of the DnaK sequence. DnaK can often be eliminated by the addition of MgATP (Rial & Ceccarelli, 2002). We tested several MgATP conditions and found the most efficient to be a 5 mM MgATP wash during Ni^2+^-affinity chromatography (Fig S2). This wash eliminated the unresolved shoulder on P1. We next ascertained the stoichiometry and homogeneity of the peaks using SEC-MALS (Fig 2E). P1 had a measured MW of 115.7 ± 2.5 kD, consistent with a 2:1 complex (theoretical MW of 114.8 kD). P2, on the other hand, was a mixture of at least two species with MWs near a 2:1 complex and unbound Vps75 dimer, whereas P3 is mostly an unbound Vps75 dimer. Clean separation of the 2:1 complex and unbound Vps75 dimer did not occur during gel filtration, so high stringency must be used when selecting anion exchange fractions if homogeneous preparations are desired. Ultimately, we have shown that our co-expressed complex does not retain the second, weakly bound copy of Rtt109 and has a stoichiometry of 2:1.

### H3-H4 displaces Rtt109 from a 2:2 Vps75–Rtt109 complex

**Figure 3.**
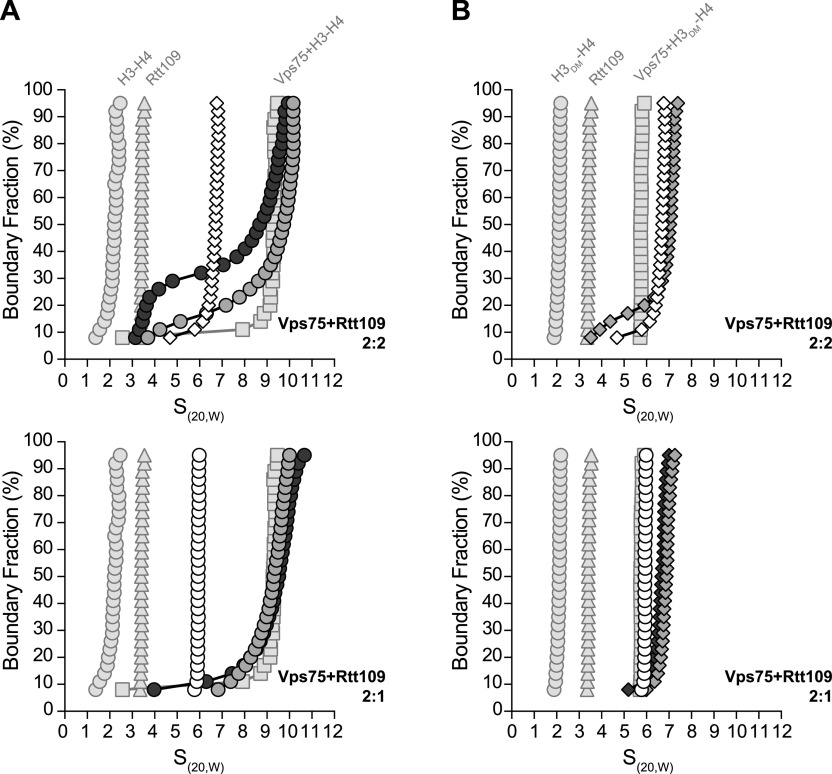
Sedimentation velocity of the Vps75–Rtt109-(H3-H4) complex. **(A, B)** van Holde–Weischet analysis of sedimentation velocity experiments of complexes with Vps75, Rtt109, and H3-H4 (A) or H3_DM_-H4 (B). Vps75 and Rtt109 were mixed 2:2 (top) or 2:1 (bottom). Vps75–Rtt109 is shown in white, and Vps75–Rtt109 and 0.5 or 1 equivalent of histones are shown in light grey and dark grey, respectively. For reference, sedimentation of H3-H4 or H3_DM_-H4 (faded circles), Rtt109 (faded triangles), and a 2:2 mix of Vps75–(H3-H4) or Vps75–(H3_DM_-H4) (faded squares) are also shown. Comparison of H3-H4 and H3_DM_-H4 sedimentation is shown in Fig S3. All experiments were carried out at 150 mM NaCl.

We next wondered if the stoichiometry of Vps75–Rtt109 complexes influenced the binding of H3-H4 substrate. We again used sedimentation velocity analysis but titrated H3-H4 against Vps75–Rtt109 that was reconstituted at either 2:2 (Fig 3A, top) or 2:1 (Fig 3A, bottom). For the 2:2 complex, addition of 0.5 equivalents of H3-H4 caused an increase in sedimentation to 10 S with a tail trailing back to 3 S. Further increasing H3-H4 to 1.0 equivalent caused a subtle decrease in sedimentation of the 10 S species, and the appearance of an obvious noninteracting 3 S species. Comparison with controls (Fig 3A, faded) identified the noninteracting species to be Rtt109. Notably, a binary complex between Rtt109 and H3-H4 is not likely, as it was not found in solution (Fig S1B and C). Addition of H3-H4 thus caused Rtt109 to be released from the 2:2 Vps75–Rtt109 complex. Rtt109 remained in the complex at 0.5 equivalents of H3-H4, as a ternary Vps75–Rtt109–(H3-H4) complex was detected. This ternary complex sedimented at 10 S, which is greater than Vps75–(H3-H4) (9.3 S) or Vps75–Rtt109 (6.7 S) (Fig 3A, top).

**Figure S3.**
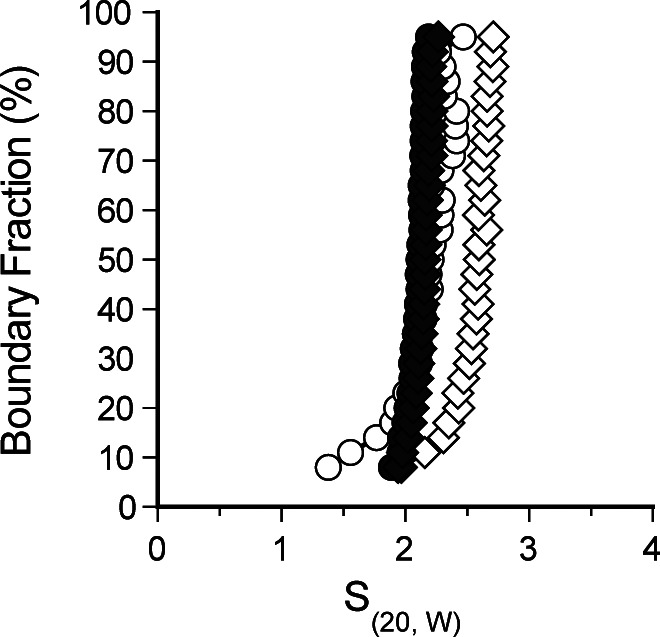
Sedimentation velocity of H3-H4 and H3_DM_-H4. van Holde–Weischet analysis of sedimentation velocity experiments of H3-H4 (white) or H3_DM_-H4 (black) at 150 mM (circles) or 300 mM (diamonds) NaCl.

One possibility is that Rtt109 is displaced by the (H3-H4)_2_ tetramer, but not by an H3-H4 dimer. We tested this by performing similar titrations with an H3 dimerization mutant (H3_DM_) (Fig 3B). The mutant contains three point mutations (C110E, L126A, and I130A) in the four-helix bundle responsible for tetramerization (Mattiroli et al, 2017). Unlike wild-type H3-H4, H3_DM_-H4 sedimentation was not influenced by ionic strength, as it is exclusively dimeric (Fig S3). Addition of H3_DM_-H4 to the 2:2 Vps75–Rtt109 complex caused a small increase in sedimentation and the appearance of a tail, comparable with that seen with wild-type H3-H4 (Fig 3B, top). This shows that one copy of Rtt109 is displaced by H3_DM_-H4. A ternary Vps75–Rtt109–(H3_DM_-H4) complex was detected at 7.1 S, which is greater than Vps75–(H3_DM_-H4) (5.8 S), or Vps75–Rtt109 (6.7 S). Taken together, these data show that H3-H4 or H3_DM_-H4 outcompete the weak binding of the second copy of Rtt109, revealing the stoichiometry of the ternary Vps75–Rtt109–(H3-H4) complex to be 2:1:1.

### 2:1:1 Vps75–Rtt109–(H3-H4) complex oligomerizes

Consistent results are obtained when titrating H3-H4 or H3_DM_-H4 against a reconstituted 2:1 Vps75–Rtt109 complex (Fig 3A and B, bottom panels). Sedimentation shifts were like those obtained with the 2:2 complex, except that the unbound Rtt109 tail was absent. There was a small tail at 1.0 equivalent, likely attributed to free H3-H4 that is unable to compete with the tight binding of the first copy of Rtt109. The shape of the sedimentation plots at the perfect 2:2:1 ratio was also notable. For H3_DM_-H4, the plot is vertical, suggesting a homogeneous 6.8 S complex (Fig 3B, bottom). The introduced H3 mutations prevented oligomerization of this complex, implying a stoichiometry of 2:2:1. By contrast, for wild-type H3-H4, the plot is sigmoidal, suggesting exchange between the 6.8 S complex and a 10 S complex (Fig 3A, bottom). This demonstrates that multiple copies of the 2:1:1 complex can dynamically associate to form a complex with a stoichiometry of 4:2:2. This association must be mediated by an H3-H3′ four-helix bundle that is mutated in H3_DM_.

**Figure 4.**
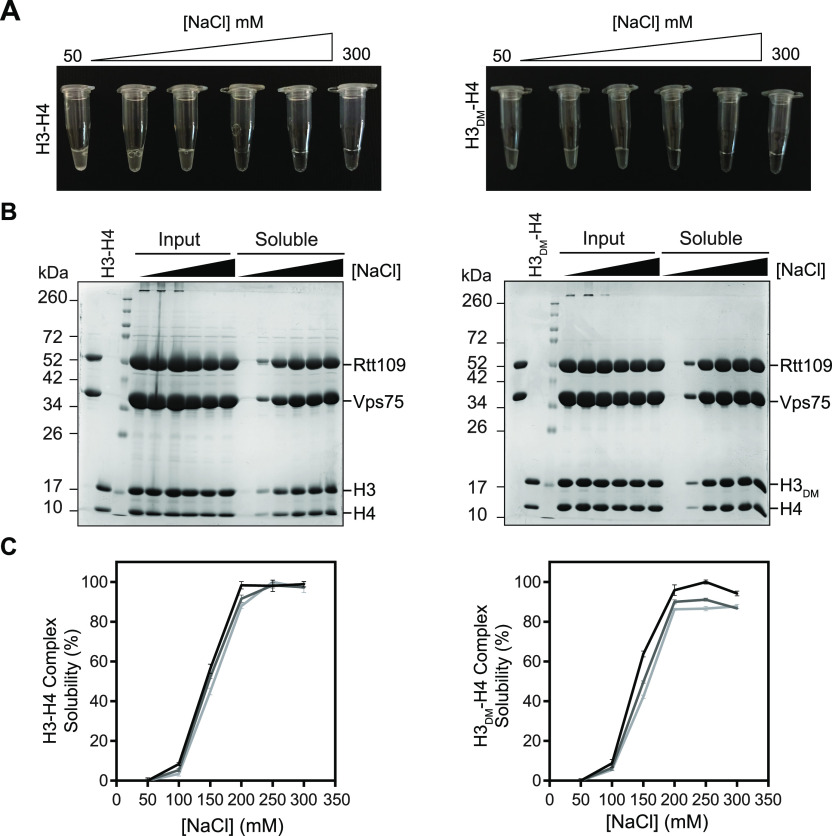
Optimizing solubility of Vps75–Rtt109–(H3-H4) complexes. Solubility of complexes containing H3-H4 (left) or H3_DM_-H4 (right) at 6 mg/ml was assayed as a function of NaCl concentration. **(A)** Image showing visual turbidity. **(B)** SDS–PAGE of samples before (Input) and after (Soluble) centrifugation. **(C)** Percent solubility of complexes at 6, 8, and 10 mg/ml based on UV spectroscopy before and after centrifugation (black, grey, and light grey, respectively). Complete data for 8 and 10 mg/ml are shown in Fig S4.

**Figure S4.**
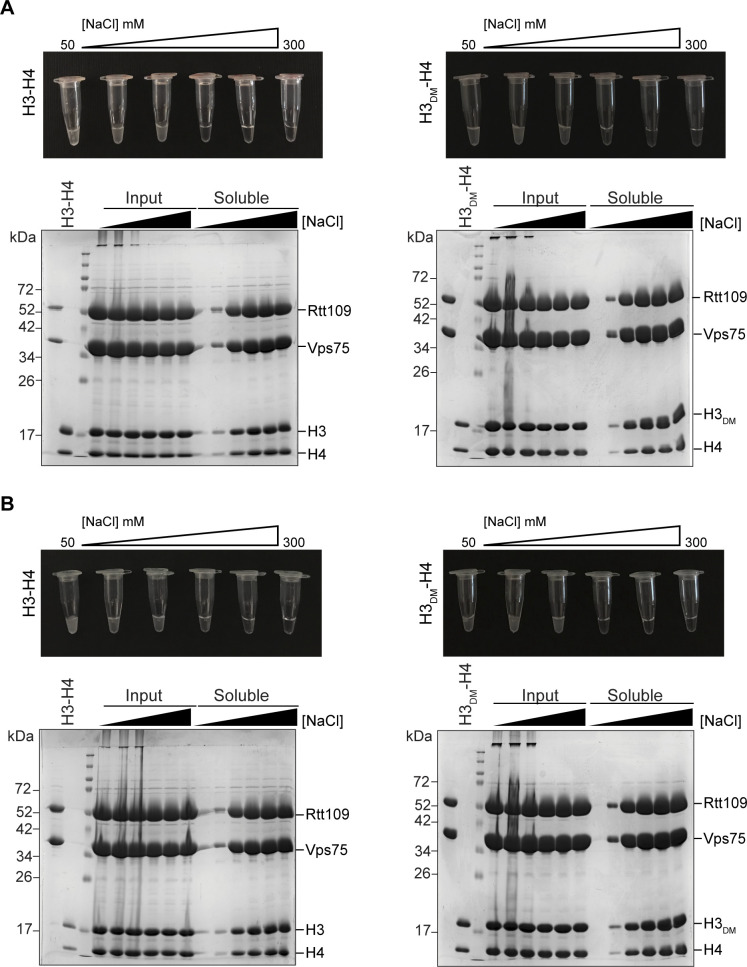
Optimizing solubility of Vps75–Rtt109–(H3-H4) complexes at 8 and 10 mg/ml. **(A, B)** Solubility of complexes containing H3-H4 (left) or H3_DM_-H4 (right) at 8 mg/ml (A) and 10 mg/ml (B) was assayed as a function of NaCl concentration. The top panel shows visual turbidity. SDS–PAGE shows samples before (input) and after (soluble) centrifugation.

We next attempted to form the 2:1:1 complex at high concentration by adding H3-H4 or H3_DM_-H4 to co-expressed Vps75–Rtt109. Initial attempts, however, were thwarted by precipitation with all samples being visually cloudy at or below 150 mM NaCl (Fig 4A). Such precipitation hindered our attempt to determine stoichiometry using SEC-MALS. As such, we prepared complexes at 6, 8, and 10 mg/ml and quantified solubility at various salt concentrations (Figs 4B and C and S4). We observe improved solubility up till 200 mM NaCl and saw only subtle differences between H3-H4 and H3_DM_-H4. These observations suggest that precipitation is due to nonspecific ionic interactions, rather than the specific oligomerization that occurs via the H3-H3′ four-helix bundle. Based on these results, we elected to perform SEC-MALS at 300 mM NaCl to ensure solubility.

**Figure 5.**
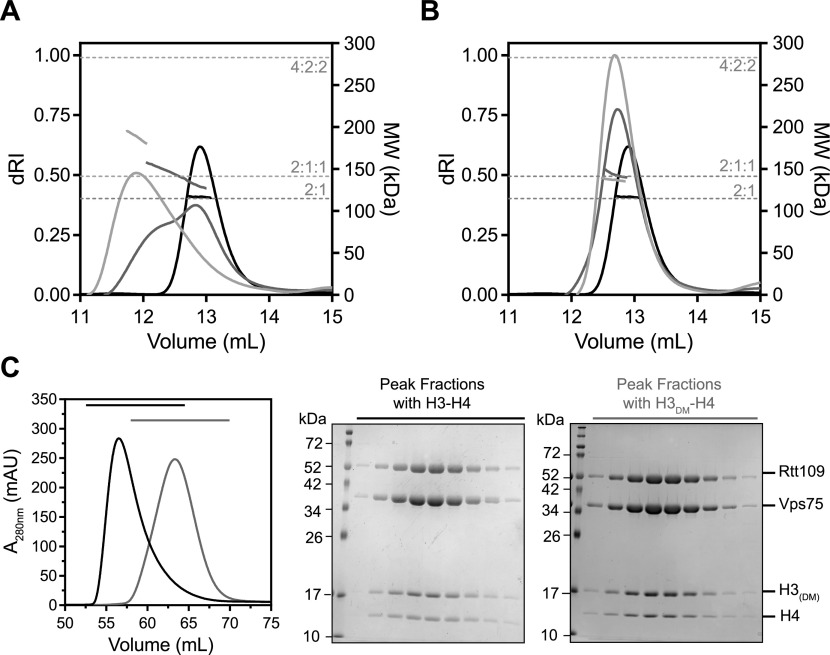
Stoichiometry and large-scale preparation of Vps75–Rtt109–(H3-H4). **(A, B)** SEC-MALS of 20 μM 2:1 Vps75–Rtt109 (black) with 20 or 40 μM (dark grey and light grey, respectively) H3-H4 (A) or H3_DM_-H4 (B). Horizontal dashed lines indicate theoretical molecular weights of labeled complexes. Molecular weights are listed in Table S2. **(C)** Gel filtration of large-scale purifications of Vps75–Rtt109–(H3-H4) (black) and Vps75–Rtt109–(H3_DM_-H4) (grey). SDS–PAGE of peak fractions.

SEC-MALS of the co-expressed 2:1 Vps75–Rtt109 complex with H3-H4 or H3_DM_-H4 gave results consistent with the sedimentation analysis. For H3-H4, mixtures eluted in a multimodal peak with a heterogeneous MW trace (Fig 5A). The MWs ranged from a theoretical 2:1:1 to 4:2:2, indicating oligomerization of a base 2:1:1 complex (Table S2). This heterogeneity was resolved when using H3_DM_-H4. For H3_DM_-H4, mixtures eluted in a single dominant peak with a homogeneous MW trace (Fig 5B). In both cases, the MW was close to a theoretical 2:1:1 complex (Table S2). Gel filtration of large-scale purifications further confirmed inclusion of the four proteins and oligomerization of the complex with wild-type H3-H4 (Fig 5C). The Vps75–Rtt109–(H3-H4) complex is thus composed of one or two copies of a stable 2:1:1 sub-complex. The two copies come together through the formation of an H3-H3′ four-helix bundle.

Table S2 Molecular weights of Vps75–Rtt109–(H3-H4) complexes measured using SEC-MALS at 300 mM NaCl.

## Discussion

We have investigated the stoichiometry of Vps75–Rtt109 using both reconstituted and co-purified complexes. We were prompted to do this as multiple crystal structures were reported showing that Vps75 can bind to Rtt109 at 2:2 or 2:1 (Su et al, 2011; Tang et al, 2011). We find that both the 2:2 and 2:1 complex can be reconstituted, depending on protein concentration and buffer ionic strength. The second copy of Rtt109 is easily lost, indicating a weaker binding affinity. This is consistent with previous analytical ultracentrifugation data, NMR, and analytical gel filtration data where a 2:2 complex is observed, albeit less occupied (Park et al, 2008; Tang et al, 2008; Danilenko et al, 2019). We further show that the 2:1 Vps75–Rtt109 complex does not oligomerize. This shows that Rtt109 binding to the Vps75 dimer interferes with Vps75 tetramer formation (Hammond et al, 2016). The Vps75 dimer has loose twofold symmetry as it is held together by head-to-tail packing of two helices, one from each chain, in a coiled coil (Tang et al, 2008). It is not immediately apparent what breaks this symmetry to limit the affinity of the second copy of Rtt109. Given that a 2:2 complex can be populated sufficiently to obtain a crystal structure (Tang et al, 2011), asymmetry is not likely driven by binding of Rtt109 or any associated steric hindrance. Rather, we suggest that the Vps75 dimer itself can switch between symmetric and asymmetric conformations. This switch may be modulated by extrinsic factors such as ionic strength or the binding of another ligand such as H3-H4.

The 2:1 Vps75–Rtt109 complex is also the most relevant for H3-H4 acetylation. We demonstrate this by showing that addition of H3-H4 to the 2:2 complex causes at least one copy of Rtt109 to be evicted. Our experiments are performed in the micromolar regime, and H3-H4 replaces the weakly bound Rtt109 at a stoichiometric ratio. This shows that the complex has a greater affinity for H3-H4 than the weakly bound Rtt109. Importantly, H3-H4 does not evict all copies of Rtt109 from the complex and a ternary Vps75–Rtt109–(H3-H4) complex forms. Given the comparable affinities between Vps75 and either H3-H4 or Rtt109 (Tsubota et al, 2008; Kolonko et al, 2010), complete Rtt109 eviction would require a vast excess of H3-H4. It would also require overlap (or at least an allosteric connection) between the Rtt109 and H3-H4 binding sites. It is possible that the distribution of Vps75 between different complexes with Rtt109, H3-H4, or both Rtt109 and H3-H4 is cell cycle dependent. Expression of both H3-H4 and Rtt109 peaks during S phase, whereas Vps75 expression remains relatively constant (Osley, 1991; Driscoll et al, 2007; Selth et al, 2009). Given the relevance of the 2:1 complex, we report an optimized protocol for its purification. This protocol is invaluable for future enzymatic and structural studies where homogeneous samples are required.

Finally, we have determined the stoichiometry and arrangement of the ternary Vps75–Rtt109–(H3-H4) complex. We could do this as we defined the optimal conditions for large-scale reconstitution of an H3-H4–bound Vps75–Rtt109 complex. We show that the 2:1 Vps75–Rtt109 complex can accommodate a single copy of an H3-H4 dimer or an (H3-H4)_2_ tetramer. The dimer-bound complex is homogeneous, whereas the tetramer-bound complex contains several species in dynamic equilibrium. Because the H3 contained mutations that prevent an H3-H3′ four-helix bundle, this equilibrium is attributed to the complex-forming dimers. The ternary complex of Vps75–Rtt109–(H3-H4) is thus composed of a 2:1:1 sub-complex that can dimerize to form a 4:2:2 complex. This dimerization is mediated by a four-helix bundle involving the two copies of H3. Our H3 mutant may also mimic the effects of another chaperone, Asf1, which prevents H3-H4 forming a tetramer (English et al, 2005).

Our study comes after work by Danilenko and colleagues characterizing Rtt109–(H3-H4) with both chaperones Vps75 and Asf1 (Danilenko et al, 2019). We consistently report that the basic unit of Vps75–Rtt109–(H3-H4) has a stoichiometry of 2:1:1. It remains to be seen if the in vivo complex contains Vps75, Asf1, or both chaperones, and all possible complexes warrant study. Our focus on the complex containing only Vps75, which has been detected in vivo, has allowed us to make several additional conclusions (Fillingham et al, 2008). By performing titrations at various ionic strengths, we show that there are two unequal binding sites for Rtt109 in the Vps75 dimer. The weaker of these sites is easily displaced by H3-H4, regardless of whether H3-H4 is a dimer or a tetramer. This is significant as it directly shows that the second copy of Rtt109 is not relevant for the Vps75-driven acetylation of either an H3-H4 dimer or tetramer. We show that the complex between Vps75, Rtt109, and H3-H4 exists in dynamic equilibrium between a 2:1:1 complex and a 4:2:2 complex. This equilibrium is driven by contacts between two copies of H3 that mediate the formation of a four-helix bundle. Four-helix bundles are common interfaces between histone dimers (Arents et al, 1991; Luger et al, 1997). This is a new insight into the arrangement of the complex that has repercussions for ongoing structural and drug discovery programs focused on this complex. These conclusions, as well as our optimized protocols for purification of relevant complexes, pave the way for mechanistic and high-resolution structural studies.

## Materials and Methods

### Protein expression and purification

*Saccharomyces cerevisiae* Vps75 and Rtt109 were individually expressed and purified as previously described (Park et al, 2008). The Vps75–Rtt109 complex was co-expressed and purified as described previously (Tang et al, 2011). Both genes were expressed from a pRSFDuet-1 vector in Rosetta (DE3) pLysS cells grown in 2xTY. Rtt109 was in multiple cloning site 1, and Vps75 was in multiple cloning site 2. Expression was induced at an OD_590nm_ of 0.7–0.8 with 0.5 mM IPTG and the cells left at 37°C overnight. 6 liters of culture was lysed by three passes through a homogenizer at 18,000 ψ. The lysis buffer contained 20 mM Tris–HCl pH 7.5, 500 mM NaCl, 5 mM BME, and 0.2 mM AEBSF with additional protease inhibitors (aprotinin [5 μg/ml], leupeptin [1 μg/ml], and pepstatin [1 μg/ml]). The soluble fraction was loaded onto a 5-ml HisTrap column (GE Healthcare) that was pre-equilibrated in 20 mM Tris–HCl pH 7.5, 300 mM NaCl, 5 mM BME, and 0.2 mM AEBSF with 15 mM imidazole. After washing to baseline, the complex was eluted using a 15–500 mM imidazole gradient over 8 column volumes (CVs). Before elution, the column was washed with 5 mM MgATP as described in Fig S2. Peak fractions from the HisTrap elution were combined and diluted 1 in 2 with 20 mM Tris–HCl pH 7.5, 5 mM BME, 0.2 mM AEBSF, and 0.5 mM EDTA. The diluted sample was loaded onto a MonoQ 10/100 GL column (GE Healthcare) pre-equilibrated in 20 mM Tris–HCl pH 7.5, 150 mM NaCl, 5 mM BME, 0.2 mM AEBSF, and 0.5 mM EDTA. After washing to baseline, the complex was eluted using a 150–1,000 mM NaCl gradient over 12 CVs. Purification was monitored using 15% SDS–PAGE stained with Coomassie Blue. When required, gels were quantified using Image Lab 1.1.0.04. The total intensity of each lane was set to 100%. In-gel digestion and mass spectrometry were performed using standard protocols by the Institutional Mass Spectrometry Core Laboratory at the University of Texas Health Science Center, San Antonio.

Unfolded *Xenopus laevis* wild-type H3, mutant H3_DM_ (C110E, L126A, and I130A), and H4 were purchased from the Histone Source at Colorado State University. They were refolded using standard protocols (Dyer et al, 2003).

### Sedimentation velocity analysis

Proteins were dialyzed into 20 mM Tris pH 7.5, 150 mM or 300 mM NaCl, and 0.5 mM TCEP. Mixtures were formed relative to 7.5 μM Vps75 (calculated with monomer extinction coefficient) (Figs 1 and 3), 7.5 μM Rtt109 (Fig S1), and 22.5 μM H3-H4 or H3_DM_-H4 (calculated with the H3-H4 dimer extinction coefficient) (Fig S3). Sedimentation velocity experiments were performed using a Beckman Coulter Optima XL-A or XL-I analytical ultracentrifuge. Samples were placed in standard epon 2-channel centerpiece cells. Sedimentation was then monitored using either absorbance or intensity modes at 229 or 280 nm at 20°C. Data were collected using speeds of 30,000, 35,000, 45,000, or 50,000 rpm with either an An60Ti or An50Ti rotor. Partial specific volumes were determined using UltraScan3 version 2.0. Time invariant and radial invariant noise was subtracted from the sedimentation velocity data by two-dimensional spectrum analysis, followed by genetic algorithm refinement and Monte Carlo analysis (Demeler & Brookes, 2008). Sedimentation coefficient distributions G(s) were obtained with enhanced van Holde–Weischet analysis (Demeler et al, 1997). Calculations were performed on the UltraScan LIMS cluster at the Bioinformatics Core Facility, University of Texas Health Science Center, San Antonio; and the Lonestar cluster at the Texas Advanced Computing Center, supported by NSF Teragrid Grant #MCB070038.

### SEC-MALS

For analysis of Vps75–Rtt109 only (Fig 1C), proteins were dialyzed into 20 mM Tris pH 7.5, 150 mM NaCl, and 1 mM TCEP. Mixtures were formed relative to 10 μM Vps75. Rtt109 alone was also prepared at 10 μM. 100 μl of each sample was injected over a Superdex 200 10/300 GL column using an ÄKTA purifier HPLC system (GE Healthcare) at 0.3 ml/min. This system was directly connected to a Dawn Heleos II multi-angle light scattering instrument and a refractive index detector (Wyatt Technologies). Data were analyzed with ASTRA 5.

For analysis of Vps75–Rtt109 anion exchange peaks (Fig 2E) and Vps75–Rtt109–(H3-H4) complexes (Fig 5A), samples were dialyzed into to 20 mM Tris–HCl pH 7.5, 300 mM NaCl, and 0.5 mM TCEP. Samples were concentrated to 20 μM or mixtures formed relative to 20 μM Vps75. 100 μl of each sample was injected over a Superdex 200 Increase 10/300 GL column using an ÄKTA pure HPLC system (GE Healthcare) at 0.75 ml/min. This system was directly connected to a miniDAWN TREOS and Optilab T-rEX refractive index detectors (Wyatt Technologies). Data were analyzed with ASTRA 7. Detailed protocols are published (Sarkar et al, 2020).

### Solubility analysis

Concentrated stocks of 2:1 Vps75–Rtt109, H3-H4, and H3_DM_-H4 were prepared in 20 mM Tris–HCl pH 7.5, 300 mM NaCl, and 0.5 mM TCEP. Vps75–Rtt109 and histones were then combined 1:1 and diluted to 6, 8, or 10 mg/ml. The dilution buffer was adjusted to give a final NaCl concentration of 50–300 mM in 50 mM increments. Aliquots were taken before (input) and after (soluble) 2-min centrifugation at 8,800*g* and analyzed by 15% SDS–PAGE stained with Coomassie Blue. Triplicate UV spectra (190–850 nm) were also measured before and after centrifugation using a NanoDrop One/One^C^ UV-Vis Spectrophotometer. Mean data were plotted using GraphPad Prism 8 showing 95% confidence intervals.

#### Large-scale gel filtration

Vps75–Rtt109-(H3-H4) and Vps75–Rtt109–(H3_DM_-H4) were formed by mixing co-expressed Vps75–Rtt109 and histone 1:1. Samples were then injected over a Superdex 200 16/60 prep grade column (GE Healthcare) pre-equilibrated in 20 mM Tris–HCl pH7.5, 300 mM NaCl, and 0.5 mM TCEP. Peak fractions were run on 15% SDS–PAGE and visualized with Coomassie Blue.

## Supplementary Material

Reviewer comments

## References

[bib1] Albaugh BN, Kolonko EM, Denu JM (2010) Kinetic mechanisms of histone acetyltransferase and chaperone complex Rtt109-Vsp75. Biochemistry 49: 6375–6385. 10.1021/bi100381y 20560668 PMC2917178

[bib2] Arents G, Burlingame RW, Wang BIC, Love WE, Moudrianakis EN (1991) The nucleosomal core histone octamer at 3.1 Å resolution: A tripartite protein assembly and a left-handed superhelix. Proc Natl Acad Sci U S A 88: 10148–10152. 10.1073/pnas.88.22.10148 1946434 PMC52885

[bib3] Bannister AJ, Kouzarides T (2011) Regulation of chromatin by histone modifications. Cell Res 21: 381–395. 10.1038/cr.2011.22 21321607 PMC3193420

[bib4] Berndsen CE, Tsubota T, Linder SE, Lee S, Holton JM, Kaufman PD, Keck JL, Denu JM (2008) Molecular functions of the histone acetyltransferase chaperone complex Rtt109-Vps75. Nat Struct Mol Biol 15: 948–956. 10.1338/nsmb.1459 19172748 PMC2678805

[bib5] Bowman A, Hammond CM, Stirling A, Ward R, Shang W, El-Mkami H, Robinson DA, Svergun DI, Norman DG, Owen-Hughes T (2014) The histone chaperones Vps75 and Nap1 form ring-like, tetrameric structures in solution. Nucleic Acids Res 42: 6038–6051. 10.1093/nar/gku232 24688059 PMC4027167

[bib6] Chen CC, Carson JJ, Feser J, Tamburini B, Zabaronick S, Linger J, Tyler JK (2008) Acetylated lysine 56 on histone H3 drives chromatin assembly after repair and signals for the completion of repair. Cell 134: 231–243. 10.1016/j.cell.2008.06.035 18662539 PMC2610811

[bib7] Creyghton MP, Cheng AW, Welstead GG, Kooistra T, Carey BW, Steine EJ, Hanna J, Lodato MA, Frampton GM, Sharp PA, (2010) Histone H3K27ac separates active from poised enhancers and predicts developmental state. Proc Natl Acad Sci U S A 107: 21931–21936. 10.1073/pnas.1016071107 21106759 PMC3003124

[bib8] Dahlin JL, Kottom T, Han J, Zhou H, Walters MA, Zhang Z, Limper H (2014) Pneumocystis jirovecii Rtt109 , a novel drug target for pneumocystis pneumonia in immunosuppressed humans. Antimicrob Agents Chemother 58: 3650–3659. 10.1128/AAC.02637-14 24733475 PMC4068529

[bib9] Danilenko N, Lercher L, Kirkpatrick J, Gabel F, Codutti L, Carlomagno T (2019) Histone chaperone exploits intrinsic disorder to switch acetylation specificity. Nat Commun 10: 1–11. 10.1038/s41467-019-11410-7 31387991 PMC6684614

[bib10] Demeler B, Brookes E (2008) Monte Carlo analysis of sedimentation experiments. Colloid Polym Sci 286: 129–137. 10.1007/s00396-007-1699-4

[bib11] Demeler B, Saber H, Hansen JC (1997) Identification and interpretation of complexity in sedimentation velocity boundaries. Biophys J 72: 397–407. 10.1016/S0006-3495(97)78680-6 8994626 PMC1184330

[bib12] Driscoll R, Hudson A, Jackson SP (2007) Yeast Rtt109 promotes genome stability by acetylating histone H3 on lysine 56. Science 315: 649–652. 10.1126/science.1135862 17272722 PMC3334813

[bib13] Dyer PN, Edayathumangalam RS, White CL, Bao Y, Chakravarthy S, Muthurajan UM, Luger K (2003) Reconstitution of nucleosome core particles from recombinant histones and DNA. Methods Enzymol 375: 23–44. 10.1016/S0076-6879(03)75002-2 14870657

[bib14] English CM, Maluf NK, Tripet B, Churchill MEA, Jessica K (2005) ASF1 binds to a heterodimer of histones H3 and H4: A two-step mechanism for the assembly of the H3–H4 heterotetramer on DNA. Biochemistry 44: 13673–13682. 10.1021/bi051333h 16229457 PMC4445473

[bib15] Fillingham J, Suter B, Emili A, Greenblatt JF, Recht J, Allis CD, Silva AC, Keogh M-C, Stagljar I, Krogan NJ (2008) Chaperone control of the activity and specificity of the histone H3 acetyltransferase Rtt109. Mol Cell Biol 28: 4342–4353. 10.1128/MCB.00182-08 18458063 PMC2447148

[bib16] Hammond CM, Sundaramoorthy R, Larance M, Lamond A, Stevens MA, El-Mkami H, Norman DG, Owen-Hughes T (2016) The histone chaperone Vps75 forms multiple oligomeric assemblies capable of mediating exchange between histone H3-H4 tetramers and Asf1-H3-H4 complexes. Nucleic Acids Res 44: 6157–6172. 10.1093/nar/gkw209 27036862 PMC5291247

[bib17] Keck KM, Pemberton LF (2011) Interaction with the histone chaperone Vps75 promotes nuclear localization and HAT activity of Rtt109 in vivo. Traffic 12: 826–839. 10.1111/j.1600-0854.2011.01202.x 21463458 PMC3115401

[bib18] Kolonko EM, Albaugh BN, Lindner SE, Chen Y, Satyshur KA, Arnold KM, Kaufman PD, Keck JL, Denu JM (2010) Catalytic activation of histone acetyltransferase Rtt109 by a histone chaperone. Proc Natl Acad Sci U S A 107: 20275–20280. 10.1073/pnas.1009860107 21057107 PMC2996700

[bib19] Kuo YM, Henry RA, Huang L, Chen X, Stargell LA, Andrews AJ (2015) Utilizing targeted mass spectrometry to demonstrate Asf1-dependent increases in residue specificity for Rtt109-Vps75 mediated histone acetylation. PLoS One 10: 1–18. 10.1371/journal.pone.0118516 PMC4364511 25781956

[bib20] Li Q, Zhou H, Wurtele H, Davies B, Horazdovsky B, Verreault A, Zhang Z (2008) Acetylation of histone H3 lysine 56 regulates replication- coupled nucleosome assembly. Cell 134: 244–255. 10.1016/j.cell.2008.06.018 18662540 PMC2597342

[bib21] Luger K, Mäder AW, Richmond RK, Sargent DF, Richmond TJ (1997) Crystal structure of the nucleosome core particle at 2.8 Å resolution. Nature 389: 251–260. 10.1038/38444 9305837

[bib22] Mattiroli F, Gu Y, Yadav T, Balsbaugh JL, Harris MR, Findlay ES, Liu Y, Radebaugh CA, Stargell LA, Ahn NG, (2017) DNA-mediated association of two histone-bound complexes of yeast Chromatin Assembly Factor-1 (CAF-1) drives tetrasome assembly in the wake of DNA replication. Elife 6: e22799 10.7554/eLife.22799.001 28315523 PMC5404915

[bib23] McBryant SJ, Peersen OB (2004) Self-association of the yeast nucleosome assembly protein 1. Biochemistry 43: 10592–10599. 10.1021/bi035881b 15301556

[bib24] McGinty RK, Tan S (2015) Nucleosome structure and function. Chem Rev 115: 2255–2273. 10.1021/cr500373h 25495456 PMC4378457

[bib25] Osley M (1991) The regulation of histone synthesis in the cell cycle. Annu Rev Biochem 60: 827–861. 10.1146/annurev.biochem.60.070191.004143 1883210

[bib26] Park Y-J, Sudhoff KB, Andrews AJ, Stargell LA, Luger K (2008) Histone chaperone specificity in Rtt109 activation. Nat Struct Mol Biol 15: 957–964. 10.1038/nsmb.1480 19172749 PMC2680711

[bib27] Radovani E, Cadorin M, Shams T, El-Rass S, Karsou AR, Kim HS, Kurat CF, Keogh MC, Greenblatt JF, Fillingham JS (2013) The carboxyl terminus of Rtt109 functions in chaperone control of histone acetylation. Eukaryot Cell 12: 654–664. 10.1128/EC.00291-12 23457193 PMC3647767

[bib28] Recht J, Tsubota T, Tanny JC, Diaz RL, Berger JM, Zhang X, Garcia BA, Shabanowitz J, Burlingame AL, Hunt DF, (2006) Histone chaperone Asf1 is required for histone H3 lysine 56 acetylation, a modification associated with S phase in mitosis and meiosis. Proc Natl Acad Sci U S A 103: 6988–6993. 10.1073/pnas.0601676103 16627621 PMC1459006

[bib29] Rial DV, Ceccarelli EA (2002) Removal of DnaK contamination during fusion protein purifications. Protein Expr Purif 25: 503–507. 10.1016/S1046-5928(02)00024-4 12182832

[bib30] Roh TY, Cuddapah S, Zhao K (2005) Active chromatin domains are defined by acetylation islands revealed by genome-wide mapping. Genes Dev 19: 542–552. 10.1101/gad.1272505 15706033 PMC551575

[bib31] Sarkar P, Akhavantabib N, D’Arcy S (2020) Comprehensive analysis of histone-binding proteins with multi-angle light scattering. Methods 10.1016/j.ymeth.2020.01.014 PMC7381358 31988003

[bib32] Schneider J, Bajwa P, Johnson FC, Bhaumik SR, Shilatifard A (2006) Rtt109 is required for proper H3K56 acetylation: A chromatin mark associated with the elongating RNA polymerase II. J Biol Chem 281: 37270–37274. 10.1074/jbc.C600265200 17046836

[bib33] Selth L, Svejstrup JQ (2007) Vps75, a new yeast member of the NAP histone chaperone. J Biol Chem 282: 12358–12362. 10.1074/jbc.C700012200 17344218

[bib34] Selth LA, Lorch Y, Ocampo-Hafalla MT, Mitter R, Shales M, Krogan NJ, Kornberg RD, Svejstrup JQ (2009) An rtt109-independent role for Vps75 in transcription-associated nucleosome dynamics. Mol Cell Biol 29: 4220–4234. 10.1128/mcb.01882-08 19470761 PMC2715805

[bib35] Shahbazian MD, Grunstein M (2007) Functions of site-specific histone acetylation and deacetylation. Annu Rev Biochem 76: 75–100. 10.1146/annurev.biochem.76.052705.162114 17362198

[bib36] Su D, Hu Q, Zhou H, Thompson JR, Xu RM, Zhang Z, Mer G (2011) Structure and histone binding properties of the Vps75-Rtt109 chaperone-lysine acetyltransferase complex. J Biol Chem 286: 15625–15629. 10.1074/jbc.C111.220715 21454705 PMC3091171

[bib37] Tang Y, Meeth K, Jiang E, Luo C, Marmorstein R (2008) Structure of Vps75 and implications for histone chaperone function. Proc Natl Acad Sci U S A 105: 12206–12211. 10.1073/pnas.0802393105 18723682 PMC2527890

[bib38] Tang Y, Holbert MA, Delgoshaie N, Wurtele H, Guillemette B, Meeth K, Yuan H, Drogaris P, Lee EH, Durette C, (2011) Structure of the Rtt109-AcCoA/Vps75 complex and implications for chaperone-mediated histone acetylation. Structure 19: 221–231. 10.1016/j.str.2010.12.012 21256037 PMC3050538

[bib39] Tsubota T, Berndsen CE, Erkmann JA, Smith CL, Freitas MA, Denu JM, Kaufman PD (2008) Histone H3-K56 acetylation is catalyzed by histone chaperone-dependant complexes. Mol Cell 25: 703–712. 10.1016/j.molcel.2007.02.006 PMC1853276 17320445

[bib40] Wurtele H, Tsao S, Lépine G, Mullick A, Tremblay J, Drogaris P, Lee EH, Thibault P, Verreault A, Raymond M (2010) Modulation of histone H3 lysine 56 acetylation as an antifungal therapeutic strategy. Nat Med 16: 774–780. 10.1038/nm.2175 20601951 PMC4108442

