# Supplementary material for: Stoichiometry of Rtt109 complexes with Vps75 and histones H3-H4
Source: Life Sci Alliance. 2020 Sep 10;3(11):e202000771. doi: 10.26508/lsa.202000771 (PMC7494816; doi:10.26508/lsa.202000771)
Supplement: Supplementary file 1 [file LSA-2020-00771_TableS1.docx]

**Table S1: Summary of Sedimentation Velocity Samples**

| Sample | Mixture  Molar Ratio | [NaCl]  (nM) | Figure |
| --- | --- | --- | --- |
| Vps75 | **-** | 150 | 1A |
| Vps75-Rtt109 | 2:1  2:2 | 150  150 | 1A, 3A-B *bottom*  1A, 3A-B *top* |
| Vps75 | **-** | 300 | 1B |
| Vps75-Rtt109 | 2:1  2:2 | 300  300 | 1B  1B |
| Rtt109 | - | 150 | S1A-C, 3A-B |
| Rtt109 | - | 300 | S1A |
| Rtt109-(H3-H4) | 1:0.5  1:1  1:2 | 150  150  150 | S1B  S1B  S1B |
| Rtt109-(H3_DM_-H4) | 1:0.5  1:1  1:2 | 150  150  150 | S1C  S1C  S1C |
| H3-H4 | - | 150 | S3, 3A |
| H3-H4 | - | 300 | S3 |
| H3_DM_-H4 | - | 150 | S3, 3B |
| H3_DM_-H4 | - | 300 | S3 |
| Vps75 -(H3-H4) | 2:2 | 150 | 3 |
| Vps75- (H3_DM_-H4) | 2:2 | 150 | 3 |
| Vps75-Rtt109-(H3-H4) | 2:2:1  2:2:2 | 150  150 | 3A *top*  3A *top* |
| Vps75-Rtt109-(H3-H4) | 2:1:1  2:1:2 | 150  150 | 3A *bottom*  3A *bottom* |
| Vps75-Rtt109-(H3_DM_-H4) | 2:2:1 | 150 | 3B *top* |
| Vps75-Rtt109-(H3_DM_-H4) | 2:1:1  2:1:2 | 150  150 | 3B *bottom*  3B *bottom* |
