# Supplementary material for: Stoichiometry of Rtt109 complexes with Vps75 and histones H3-H4
Source: Life Sci Alliance. 2020 Sep 10;3(11):e202000771. doi: 10.26508/lsa.202000771 (PMC7494816; doi:10.26508/lsa.202000771)
Supplement: Supplementary file 2 [file LSA-2020-00771_TableS2.docx]

**Table S2: MWs of Vps75-Rtt109-(H3-H4) Complexes measured using SEC-MALS at 300 mM NaCl**

| Sample | Mixture Molar Ratio | Observed MW (± SE) (kDa) | Theoretical Complex | Theoretical MW  (kDa) |
| --- | --- | --- | --- | --- |
| Vps75-Rtt109 | 2:1 | 115.2 ± 1.3 | 2:1 | 114.8 |
| Vps75-Rtt109-(H3-H4) | 2:1:1 | *heterogeneous* | *mix of 2:1:1 and 4:2:2* | |
| Vps75-Rtt109-(H3-H4) | 2:1:2 | *heterogeneous* | *-* | |
| Vps75-Rtt109-(H3_DM_-H4) | 2:1:1 | 143.2 ± 0.4 | 2:1:1 | 141.4 |
| Vps75-Rtt109-(H3_DM_-H4) | 2:1:2 | 137.1 ± 0.4 | 2:1:1 | 141.4 |
